# The Opinions of Specialists in Obstetrics and Gynecology on the Indications for Pregnancy Termination in Poland—A Preliminary Cross-Sectional Study

**DOI:** 10.3390/ijerph191912578

**Published:** 2022-10-01

**Authors:** Kornelia Zaręba, Valentina Lucia La Rosa, Stanisław Wójtowicz, Ewelina Kołb-Sielecka, Jolanta Banasiewicz, Michał Ciebiera, Grzegorz Jakiel

**Affiliations:** 1 Department of Obstetrics and Gynecology, College of Medicine and Health Sciences (CMHS), United Arab Emirates University (UAEU), Al Ain 17666, United Arab Emirates; 2 Unit of Psychodiagnostics and Clinical Psychology, University of Catania, 95124 Catania, Italy; 3 Department of Medical Psychology and Medical Communication, Medical University of Warsaw, 02-091 Warsaw, Poland; 4 First Department of Obstetrics and Gynecology, Center of Postgraduate Medical Education, 01-813 Warsaw, Poland; 5 Second Department of Obstetrics and Gynecology, Center of Postgraduate Medical Education, 01-813 Warsaw, Poland

**Keywords:** abortion, physician attitude, abortion law

## Abstract

The physician’s decision concerning pregnancy termination is influenced by a number of factors. The study aimed at obtaining the opinions of obstetricians and gynecologists with regard to the indications for pregnancy termination, the readiness to perform the procedure personally and the assessment of the determinants thereof. The survey study was conducted between 1 January 2020 and 31 December 2021 among physicians who performed diagnostic prenatal ultrasonography. A considerable majority of physicians participating in the study did not approve of termination without medical indications (62.5%). A marked majority of them considered the following cases as indications for pregnancy termination: severe fetal defects (90%), lethal defects (91.5%) and a disease threatening maternal life (91.5%). A small group of physicians declared that they were ready to perform a termination without medical indications (12.5%). However, they were ready to perform a pregnancy termination personally in cases of threat to maternal life (77.5%), severe fetal defects (75%), lethal fetal defects (75%) and a pregnancy being a result of rape (75%). No statistical significance was observed with regard to the influence of the respondents’ sex, the fact of having children or the workplace on the issue of indications for pregnancy termination. It seems justified to develop case-centered counseling concerning abortion, based on specialists in perinatology, law and ethics, especially in countries with more restrictive abortion law or strongly religious societies.

## 1. Introduction

The decision concerning pregnancy termination is influenced by a number of factors, encompassing the law and religion of the country, as well as personal factors regarding the personnel participating in the procedures [1,2,3]. Importantly, the physician’s personal beliefs and willingness to perform the procedure is another, equally significant factor, which limits access to the procedure in many locations [4,5]. The viewpoint of the physicians is affected by religion, fear of social stigma, lack of medical knowledge and widely understood fear related to the performance of the procedure and its legal consequences [6,7,8].

It seems of importance to mention the issue of the conscience clause. The conscience clause is the right to refuse to perform a medical procedure because of the moral or religious beliefs of the medical personnel [9]. It was developed to protect the autonomy of an individual and moral pluralism in the society [10]. According to research, some physicians invoke the conscience clause for fear of being labeled an “abortionist”, which may constitute a problem in terms of their future professional career or perception by the society [8]. The law applicable in the country is the main determinant of the possibility of performing a termination due to the conscience clause [2]. Turk et al., reported that 19% of gynecologists would be ready to perform terminations if the law permitted them, while only 8% of the respondents were against the idea of performing a termination personally. A total of 54% of the respondents reported restrictions related to terminations both in private practice and in hospitals [2]. The main barriers regarding the performance of terminations reported by the authors were: the region of the country (practice in the western part of the country), logistic barriers and practice restrictions [2].

### Background

In Poland, abortion law has been dynamically changing with a tendency toward becoming tightened. Below, we are presenting an overview on how abortion law has been changing in Poland:

1932—The procedure was legal in case of threat to maternal health or when the pregnancy was a result of an unlawful act (Art. 32 of the Criminal Code as of 1932).

27 April 1956—The procedure was legal in three cases:

- pregnancy termination was medically indicated due to the health status of the fetus or the pregnant woman;
- if there was a justified suspicion that the pregnancy occurred as a result of an unlawful act, such as rape;
- due to the difficult living conditions of the pregnant woman (Journal of Acts dated from 1956, no 12, item 61).

7 January 1993—The Act about Family Planning, Protection of the Human Fetus, and Conditions for Pregnancy Termination (Journal of Acts no. 17, item 78, as amended) was passed. It permitted terminating a pregnancy on medical grounds indicated by the physician and the opinion of the pregnant woman in three instances:

- the pregnancy poses a direct threat to maternal health or life;
- prenatal screening or other medical evidence indicate a high probability of a severe and irreversible fetal anomaly or an incurable life-threatening disease;
- there is a reason to believe that the pregnancy is a result of an unlawful act [11].

The Constitutional Tribunal in Poland banned abortion due to fetal defects, stating that it was unconstitutional, at the end of 2020. The statement issued by the Constitutional Tribunal confirmed the necessity to protect human life from the moment of conception [12]. A tendency toward an even more intense radicalization of the abortion law is still dominant in Poland. A motion was submitted to the lower house of the Polish Parliament, which included a complete ban on abortion and 5–25 years of prison to punish a physician who would perform a pregnancy termination or who would facilitate the performance of such a procedure. However, the motion was rejected at the first reading [13]. Due to the changes in the abortion law, an increasing frequency of terminations related to the deterioration or threat to maternal health is observed in hospitals [14]. Some cases refer to the threat to the mental health of the mother and an increased risk of a mental disease in the mother linked to the continuation of a pregnancy with lethal or severe and irreversible fetal defects. The current situation is a subject of numerous debates concerning the moral and rational aspects of the procedure [14].

Currently, all women in Poland have access to prenatal diagnostics refunded by the state (ultrasound examination in the first, second and third trimester, and genetic tests in the event of indications for their implementation). We do not have accurate statistics in this area because some examinations are carried out in private offices and are not included in the statistics. The identified fetal defects and genetic defects were the basis for qualification for pregnancy termination.

The present paper depicts the opinion of Polish specialists in obstetrics and gynecology in terms of the legality of pregnancy termination and factors, which may influence their decisions. The study began in January 2020. In the meantime, the Polish abortion law became more strict, so now, abortion is illegal in cases of severe and irreversible fetal defects. The purpose of the study was to assess whether gynecologists/obstetricians supported abortion and for what indications, when they would be willing to perform the procedure personally and whether their gender, workplace, religion and the fact of having children affected their opinions on the issue. The present study was supposed to be the opinion of a group of experts on the possibility of liberalization of the abortion law in Poland.

## 2. Materials and Methods

The present study is part of a project to determine the psychological and sociological factors influencing the work of physicians, including the opinions of the medical personnel on pregnancy termination. The first group was described in 2019 and included young physicians during their specialization course in obstetrics and gynecology. This study presents the second study group, which only included physicians performing diagnostic prenatal ultrasonography. It includes the analysis of questionnaires completed by gynecologists who perform fetal diagnostics. Physicians performing prenatal ultrasonographic diagnostics of the fetus are a group of gynecologists who usually have the most extensive knowledge about the possible complications and prognosis concerning the life and health in cases of severe fetal defects, maternal diseases and potential medical complications related to the delay of pregnancy termination. The majority of them are specialized in perinatology. Therefore, we decided to analyze a group of individuals who were professionally involved in this issue and asked their opinions about the indications for pregnancy termination in terms of other factors. The total number of obstetrics and gynecology specialists with sub-specialization in perinatology is below 100 in Poland. According to FMF data, there are 473 doctors with a valid Fetal Medicine Foundation (FMF) Certificate. However, not all of them are specialists in obstetrics and gynecology [15].

The study was conducted from 1 January 2020 to 31 December 2021. The physicians were recruited prospectively. They were asked by a physician conducting the study to complete an anonymous questionnaire in private.

The inclusion criteria were as follows: consent to participate in the study and the possession of a certificate authorizing the performance of prenatal screening tests and performing these tests in daily clinical practice. The exclusion criteria were: lack of consent to participate in the study and lack of appropriate authorization to perform fetal ultrasound. In Poland, prenatal diagnostics should be carried out according to the guidelines of the Ultrasonography Section of the Polish Society of Gynecologists and Obstetricians. Due to the necessity of calculating the risk of selected trisomies, the physician should have a valid certificate issued by the British Fetal Medicine Foundation (FMF) [16,17].

A total of 62 questionnaires were distributed, and 40 of them were returned (64.5%).

### 2.1. Questionnaire

The questionnaire was developed by a team of gynecologists and psychologists after considering the purpose of the study. No standardized questionnaire measuring the exact variables of interest was available. Therefore, the assessment of the sociogeographic and other variables describing the study group was not possible with the available standardized tools. The present authors’ questionnaire allowed for the most accurate results in terms of the purpose of the study and the characteristics of the study group.

Based on the hypotheses formulated before the questionnaire was prepared, a connection was found between the world view, political views, religion and attitude toward abortion. Such hypotheses were inspired by scientific reports in the field of population studies, which also emphasized the impact of these variables on shaping the attitudes concerning abortion, particularly by the research of the Public Opinion Research Center (CBOS). The CBOS is one of the most important specialized Polish institutions conducting representative surveys of the Polish society. The center surveys the opinions of respondents on the most important social, political and economic issues. The conclusions of the CBOS population-based study indicated that world view, religion and political views were related to attitudes toward abortion.

The authors developed a questionnaire that included 32 questions grouped into four sections: general information, religion, world view and final remarks. Some answers were presented on a 5-point Likert scale (a—“I strongly agree” through e—“I strongly disagree”). Due to the lack of a standardized questionnaire, the present authors developed their own questionnaire for the needs of the study. Indications for pregnancy termination were divided according to the law applicable in the majority of European countries, i.e., social indications (no medical indications), severe and incurable fetal defects, lethal defects, threat to maternal life and trisomy 21 without other structural defects as a defect, which raises the most controversies with regard to indications for pregnancy termination [18]. Completing one questionnaire required about 10 min. Any doubts related to the questions included in the questionnaire were clarified by the researcher.

### 2.2. Statistical Analysis

A nominal scale was used to measure the majority of the variables due to the questionnaire-based character of the study. Preliminary analyses included descriptive statistics, such as the mean (age and job seniority) or numerical and percentage frequencies. Statistical software was used to generate the cross tables. Some questions were answered with the use of a Likert scale (a—“I strongly agree” through e—“I strongly disagree”), while others were multiple-choice questions. The statistical significance of differences between the distribution of results was determined with the chi-square test because we were dealing with nonparametric variables and rather small groups, which is due to the fact that it was a preliminary study. The criterion of statistical inference was set at the level of significance of *p* < 0.05. No multivariate statistics were used due to the small group size.

Analyses were conducted with IBM SPSS Statistics for Windows, Version 24.0., Armonk, NY, USA: IBM Corp. (Released 2016).

The study was approved by the Bioethics Committee (approval number 71/PB/2018).

## 3. Results

A total of 62 questionnaires were distributed, and 40 questionnaires were obtained, with a return rate of 64.5%. Subsequently, statistical analysis was performed.

### 3.1. Study Group Characteristics

The obtained data showed that the mean age of the physicians was 43.93 ± 11.07 years, with the majority being male (67.5%) (Table 1). The majority of the respondents worked at previous or current regional hospitals (70%), while 37.5% worked at university hospitals. The marked majority of the respondents lived in central Poland. The mean job seniority was 17.15 ± 10.7 years.

Most respondents were married (77.5%) and had children (82.5%). Catholic denomination was declared by the majority, i.e., 82.5% of the respondents, with 37.5% of them describing themselves as practicing Catholics. The majority of the respondents (77.5%) claimed that the society was intolerant with regard to abortion.

### 3.2. Acceptance of Indications for Abortion

A considerable majority of physicians participating in the study did not approve of termination without medical indications (62.5%) (Table 2). Severe fetal defects (90%), similar to lethal defects (91.5%) and a serious disease threatening maternal life (91.5%), were considered as indications for pregnancy termination by the marked majority of the respondents. Interestingly, considerably fewer, i.e., about 47.5% of the respondents, claimed that termination should be allowed until the end of pregnancy in cases of lethal fetal defects. Differences also occurred in cases of prenatal diagnosis of trisomy 21 without anatomic defects; 70% of the respondents considered this an indication for pregnancy termination. Only two physicians considered this an indication until the end of pregnancy, while for the majority, it was an indication up to 22 gestational weeks. Only four respondents were against pregnancy termination due to severe fetal defects, and three respondents were against any form of abortion.

The respondents who considered severe fetal defects as an indication for pregnancy termination also more commonly approved of terminations for other indications, except social ones (*p* = 0.001) (Table 3).

Analogous results were obtained in a group of respondents approving of pregnancy termination in cases of lethal fetal defects (Table 4). Statistically significant results were obtained only in cases of severe and lethal fetal defects.

Individuals approving of abortion in case of rape-related pregnancy also more commonly approved of other indications (*p* = 0.001). Similarly, individuals approving of pregnancy terminations in case of a prenatal diagnosis of trisomy 21 also more often approved of termination in case of threat to maternal health and life or pregnancy being a result of rape (*p* = 0.001).

### 3.3. Readiness to Perform Abortion Personally

A small group of physicians declared that they were ready to approve of a termination without medical indications (12.5%) (Table 5). However, the cases of threat to maternal life (77.5%), severe fetal defects (75%), lethal fetal defects (75%) and a rape-related pregnancy (75%) were viewed by a considerable majority of the respondents as indications for pregnancy termination, and they were ready to perform a pregnancy termination personally.

Interesting results were obtained in the case of questions, which concerned patient referrals to centers where pregnancy terminations could be performed. Only 52.5% of the respondents referred patients to facilities where they could undergo an abortion. Interestingly, 27.5% of the respondents provided no answer to this question, and the remaining 20% did not refer patients to centers where they could undergo a termination.

### 3.4. The Influence of the Respondents’ Sex, Place of Work and the Fact of Having Children on the Opinion on the Indications for Pregnancy Termination

The analysis revealed no statistical significance with regard to the influence of the respondents’ sex, the fact of having children or the place of work on the issue of indications for pregnancy termination. The data indicated that men from the study population were slightly less strict in cases of severe and lethal fetal defects being the causes of terminations. A higher level of approval by men (32% vs. 19%) was also observed for pregnancy termination up to 22 weeks of gestation in case of a rape-related pregnancy. Men also more commonly approved of pregnancy termination in the absence of medical indications (42.5%). Similarly, all men and 18.8% of women approved of terminations in case of a serious maternal disease. Men were also less strict in cases of trisomy 21 diagnosis; a total of 17.4% of men and 50% of women were against termination. Moreover, men were more frequently ready to perform the procedure personally, which was declared by 87% in case of confirmed medical indications.

Physicians working at university hospitals more commonly considered a serious maternal disease as an indication for possible pregnancy termination, also at later stages of pregnancy. Only 6.7% of the respondents were against it. Similarly, 26.7% of physicians working at university hospitals and 34.8% of those working at other types of hospitals were against terminations related to trisomy 21. It is particularly interesting that the readiness to perform the procedure personally was considerably more often declared by physicians who did not work at university hospitals.

The viewpoint of the respondents who had children was much stricter with regard to indications for pregnancy termination, which was an important and interesting observation. However, 71.4% of individuals approved of access to pregnancy termination until the end of gestation in cases of severe, incurable and lethal fetal defects. Similarly, 42.9% of childless respondents vs. 3.1% of those who had children approved of pregnancy termination until the end of a rape-related pregnancy. With regard to a serious maternal disease, it was considered a possible indication for pregnancy termination until the end of gestation by 85.7% of childless respondents. Moreover, childless physicians more commonly declared they were ready to perform a termination without medical indications.

### 3.5. Opinion on Indications for Pregnancy Termination in Case of a Disease of One’s Child or Partner

In case of a potential lethal defect diagnosed in one’s own child, 72.5% of the respondents would agree, while 10% would be against pregnancy termination. Those who approved of termination in case of lethal defects would also more often agree to terminate a pregnancy of their own offspring with confirmed lethal defects. The correlation was statistically significant (*p* = 0.001) (Table 6).

The majority of physicians approving of termination in case of lethal fetal defects would agree to such a procedure if their own offspring had such defects, while all the respondents who did not approve of termination would not agree to have it performed even in the case of their own child. Analogous results were obtained with regard to a threat to maternal life (Table 7).

## 4. Discussion

The issue of indications for pregnancy termination and abortion law is characterized by enormous dynamics worldwide. In the case of Poland, the current political situation has become a significant determinant. The example of Ireland showed that liberalization of the abortion law could be implemented, while in Poland, the law has been radicalized [12,19].

The present study showed that the opinions of the study population of gynecologists and obstetricians concerning indications for abortion were less strict than the law applicable in Poland, and the majority of physicians declared to be ready to perform such a procedure personally (about 75%). No approval for the procedure of abortion was expressed more commonly in cases of no medical indications. However, in cases of threat to maternal life (77.5%), severe fetal defects (75%), lethal fetal defects (75%) and a rape-related pregnancy (75%), a considerable majority of the respondents claimed they were willing to perform pregnancy termination. A study conducted in Mexico and Bolivia, similarly to the present one, showed that healthcare workers were not willing to perform pregnancy terminations for social indications and believed that it was due to the fact that effective contraception was not used [20]. A study conducted in Wisconsin (USA) showed that 62% of the respondents approved of abortion. The highest rates of approval were reported for obstetricians, gynecologists and family doctors, i.e., those who participated in the procedure of abortion [21]. In the USA, it is legal for family doctors to prescribe abortion pills. A study conducted in the students of medicine in Thailand revealed that the majority of students declared they were willing to perform an abortion in case of a threat to maternal or fetal life, or if the pregnancy was a result of rape [22]. A small group declared that they were ready to perform a termination without medical indications. Analogous results were obtained in the present study, i.e., a small group of physicians declared that they would be ready to approve of a termination without medical indications (12.5%). The most liberal views on abortion are probably observed in the Benelux countries. A study performed in the medical personnel participating in the procedure of pregnancy termination in Belgium showed that late terminations due to lethal defects were approved of by 100% of the respondents, and 95.6% of them approved of termination due to severe fetal defects [23]. With regard to social indications, only 13.2% approved of the possibility of late terminations. Pregnancy termination in case of severe fetal defects was approved of by physicians more commonly than by the remaining medical personnel (68.1% vs. 53.2%). Willingness to help or personally perform a pregnancy termination in case of a severe fetal disease was reported by 89.1% [23].

The present study showed no differences between university hospital physicians and those from other hospitals with regard to the approval of abortion. Physicians working at university hospitals more commonly approved of pregnancy termination due to a serious maternal disease, also at later stages of pregnancy. In our estimation, this may be due to the fact that those physicians were more commonly involved in the care of such patients and observed the complications related to a severe maternal disease. A study performed in Thailand showed that physicians working at tertiary facilities also more often declared readiness to perform a pregnancy termination for social indications and/or mental disease in the mother than physicians trained in regional hospitals [21]. Moreover, a study conducted by Luchetti et al., revealed that medical students from regional hospitals were more conservative with regard to indications for an abortion than students from university hospitals [24]. No detailed data were available on the reasons, or they were dependent on specific locations. This may result from the fear of being labeled an “abortionist” [24].

A study by Daniel et al., revealed that female gynecologists and medical students declared readiness to assist in abortion more commonly than men if the dispensing requirements for mifepristone were removed [25]. A study conducted in the graduates of medical schools showed that men were more commonly willing to perform terminations personally in cases of social indications [22]. Analogous results were obtained in the present study. About 42.5% of men considered social indications to be justified for pregnancy termination. Men were also more commonly ready to perform termination procedures personally, which was declared by 87% of the respondents in case of medical indications. A total of 18.8% of women were against termination in case of a serious maternal disease, while all men approved of it.

Being a parent increases sympathy and stimulates therapeutic bond to a larger extent [26]. According to our data, individuals who had children were more strict with regard to indications for pregnancy termination. Childless respondents approved of termination until the end of pregnancy in case of severe, incurable and lethal defects in the fetus (71.4%) and in case of a serious maternal disease (85.7%). The study group expressed a similar viewpoint in case of a rape-related pregnancy, with 42.9% of childless respondents vs. 3.1% of those who had children approving of termination until the end of pregnancy. They were also more commonly willing to perform the procedure personally.

Based on the data obtained in our study, 72.5% of the respondents were willing to perform pregnancy termination personally in case of a lethal defect of one’s own child, while in case of a threat to maternal life, the percentage reached 92%. The obtained results may indicate a high level of empathy of the respondents. A study conducted in Brazilian students of medicine revealed that, in their opinion, abortion might prevent mental trauma [27]. The cited study emphasized the empathy of the students who focused on the feelings of women during the abortion procedures. Seemingly, empathy may underlie the liberal viewpoint of physicians regarding the accessibility of pregnancy termination for other women compared to the attitude toward one’s own child or partner.

A study conducted in Chile showed that students of medicine and midwifery at secular universities had a more flexible attitude to the issue of abortion compared to students of Catholic universities. In case of abortion, the conscience clause would be invoked by 12% of secular and 39% of religious university students [28]. Contrarily, only the students of secular universities in Chile stated that the conscience clause should be legally sanctioned in order not to limit access to abortion [29]. A Scandinavian study carried out in the citizens of Norway revealed that the approval of abortion rights was associated with pro-secular attitudes [30]. According to the authors, attitude toward abortion was positively associated with attitude toward secularism and negatively associated with religiosity/church attendance. In the same study, religiosity had a positive effect on the acceptance of conscientious objection [30]. The present study showed no influence of religiosity on the viewpoint on abortion, even though a marked majority of the respondents declared Catholic denomination (82.5%).

The above-mentioned study by Lappena et al., conducted in the USA revealed a positive correlation between a workplace without religious affiliation and the approval of the procedure of abortion. The study showed that, apart from individual traits, institutional factors and law applicable in a specific state played a very important role [31]. The paper indicated that institutional factors might be significant, as they contributed to a situation in which physicians who personally approved of abortion did not perform the procedure in hospitals where they worked [32]. We noticed no such correlation in our study.

A question arises as to whether individuals who disapprove of some medical procedures should work in a profession in which such procedures are part of their responsibilities in numerous countries or societies [33]. Data obtained in our study indicated that only 52.5% of the respondents referred patients to facilities where they could undergo an abortion. It is difficult to identify an explicit reason for such situation; it may be due to the fear of criminal responsibility for facilitating undergoing pregnancy termination. A study conducted by O’Connor et al., revealed that 72.2% of Irish GPs stated that the conscience clause invoked by a physician should be respected, but the physician should be obliged to refer the patient to a facility where she might obtain help in terminating a pregnancy [34]. A similar viewpoint was shared by Dickens. However, physicians should not invoke the conscience clause as the final argument in order not to refer a patient to a facility where the procedure might be performed [5]. Social pressure and the fear of possible legal consequences related to abortion make physicians frequently refuse to participate in pregnancy termination, or they do not want to inform about this type of procedure [8].

A study by Power et al., described problems with the ambiguity of the applicable law and ostracism with regard to some fetal defects. Another reported problem concerned conflicts among the personnel and related to the diagnostics of fetal defects and the potential subsequent abortion [35]. The medical environment often faced the issue of legislative interference in doctor–patient relationships, particularly in the context of abortion [35]. The above-mentioned survey study carried out in Wisconsin revealed that, according to physicians, abortion-related restrictions might have a negative impact on patient care and the ability of medical institutions to attract and retain strong physician workforce. A national survey on abortion in residency training directors at US teaching hospitals revealed that 69% of the respondents experienced opposition from their co-workers with regard to indications for abortion within the previous year. The objection was mostly expressed by nurses (58%) and anesthetists (30%) [36]. Interestingly, the midwives participating in pregnancy termination procedures due to fetal defects reported emotional problems, lack of medical skills and bureaucratic problems rather than moral issues related to abortion [1,37].

A survey study conducted among 100 Roman Catholic chaplains showed that 79% of chaplains reported problems concerning specialist knowledge in medicine and ethics (when patients asked questions before making morally difficult decisions) [38]. Therefore, it seems that physicians who deal with fetal diagnostics may constitute important conceptual support in morally difficult decisions. Hence, in some populations, it may seem justifiable to develop case-centered counseling based on specialists in perinatology, law and ethics, especially in countries with more restrictive abortion law or strongly religious societies.

### 4.1. Limitations of the Study

The present study has numerous limitations resulting from such factors as a small study group and a strongly evolving abortion law applicable in the country. The study was planned to be conducted in a larger study group. Regrettably, during this period, the abortion law in Poland was tightened, and abortion became possible only in a situation of a threat to the life and health of the mother and a legally prohibited act, such as rape. By the time the abortion law was changed, we were able to gather a group of only 40 participants. We are planning a study on a larger sample and in a group of midwives working in gynecology departments and a wide group of obstetricians and gynecologists. The group is being examined in a new political situation, which may affect the results obtained. Therefore, we cannot combine the groups and report some results, e.g., information concerning the performance of termination in case of severe and incurable fetal defects, as this indication is no longer in accordance with the law, and such procedures are no longer performed. Thus, we decided to present the results as a pilot study due to their unique nature. The group of perinatologists was supposed to constitute a group of experts with the greatest knowledge in the field of defects and prognosis for the fetus.

Regrettably, due to the controversial nature of the topic and the change in the abortion law in Poland, we also had difficulty in creating the study group due to respondents’ fear of social ostracism. Despite assurances that the survey would be anonymous, some people who refused to participate feared being identified due to the small group of specialists in the field.

The study group is small, and generalizing the result over the broader population is risky, especially as it is not a random sample. Therefore, such selection criteria may affect the results. The numbers are low, which makes it difficult to fully interpret the findings stratified by important variables (age, sex, years in practice), and it may mask important differences among practicing physicians.

Most of the doctors in the present group were from the provincial capital district because that was the region with the biggest group of doctors specialized in perinatology. Moreover, those doctors were the most willing to participate in our research. The others were afraid of being recognized in their smaller communities.

Furthermore, the study was based on the present authors’ questionnaire, which may be subject to errors due to the lack of validation. In our work, we used individual questions that were not part of the scales. The questionnaire used will only include scales in the next version.

Moreover, it needs to be considered that the study was performed in a specific group, i.e., in a cultural environment in which religion has a strong influence on the society. However, the opinion of physicians with regard to indications for pregnancy termination seems to be more liberal than the religious beliefs of the respondents might suggest. The study was conducted at a time when abortion law was being changed in Poland.

### 4.2. Strengths of the Study

The present study was limited to the group of individuals experienced in the clinical practice (mean job seniority of 17.15 ± 10.7 years) who were professionally dealing with fetal diagnostics, so they possessed extensive knowledge in the area of perinatal conditions and the related prognosis. The obtained results may be treated as the voice of an independent group of experts, which is more liberal than the law applicable in Poland.

## 5. Conclusions

Based on the obtained data, it may be concluded that the viewpoint of an independent group of physicians dealing with prenatal diagnostics was opposed to the amended Polish law with regard to the issue of pregnancy termination in case of severe and incurable fetal defects, and their attitude was less strict compared to the law in the country. Therefore, in cases raising ethical questions, it seems justified to develop case-centered counseling based on specialists in perinatology, law and ethics, especially in countries with more restrictive abortion law or strongly religious societies.

## Figures and Tables

**Table 1:** General description of the group.

Variable		N = 40
Age		43.93 ± 11.07
Sex	Women	17 (42.5%)
	Men	23 (57.5%)
Size of the town/city	Provincial capital	28 (70.0%)
	District capital	9 (22.5%)
	Other towns	3 (7.5%)
Province	Northern Poland	8 (20.0%)
	Southern Poland	3 (7.5%)
	Eastern Poland	3 (7.5%)
	Western Poland	1 (2.5%)
	Central Poland	22 (54%)
	No data	3 (7.5%)
Type of hospital	Teaching hospital	15 (37.5%)
	Provincial hospital	5 (12.5%)
	District hospital	11 (27.5%)
	Another hospital	7 (17.5%)
	No data	2 (5.0%)
Job seniority in gynecology		
17.15 ± 10.7	Unmarried	4 (10%)
	Married	31 (77.5%)
	Divorced	5 (12.5%)
Children	No	7 (17.5%)
	Yes	33 (82.5%)
Religion	Catholicism	33 (82.5%)
	No religious belief	7 (17.5%)

**Table 2:** Approval of pregnancy terminations in individual clinical situations.

Indications for Abortion	Until the End of Pregnancy	Up to 22 Gestational Weeks	Up to 12 Gestational Weeks	No Approval
Abortion without medical indications	2 (5.0%)	2 (5.0%)	11 (27.5%)	25 (62.5%)
Severe fetal defects	14 (35.0%)	21 (52.5%)	1 (2.5%)	4 (10.0%)
Lethal fetal defects	19 (47.5%)	17 (42.5%)	1 (2.5%)	3 (7.5%)
Rape	4 (10.0%)	10 (25.0%)	20 (50.0%)	5 (12.5%)
Serious life-threatening maternal disease	14 (35.0%)	17 (42.5%)	6 (15.0%)	3 (7.5%)
Down syndrome without anatomical anomalies	3 (7.5%)	24 (60.0%)	1 (2.5%)	12 (30.0%)

**Table 3:** Approval of pregnancy terminations in case of severe fetal defects vs. approval of other indications for terminations (N = 40).

Do You Approve of Pregnancy Termination in Case of Severe Fetal Defects?		Severe Fetal Defects	Lethal Fetal Defects	Serious Maternal Disease	Pregnancy Being a Result of Rape	Unwanted Pregnancy	Never
yes, always and until the end of pregnancy	N	12	13	12	11	2	1
	%	38.7%	37.1%	37.5%	36.7%	40.0%	20.0%
yes, until the end of 12 gestational weeks	N	0	1	1	0	0	0
	%	0%	2.9%	3.1%	0%	0%	0%
yes, up to 22 gestational weeks	N	19	21	19	19	3	0
	%	61.3%	60.0%	59.4%	63.3%	60.0%	0%
no	N	0	0	0	0	0	4
	% z	0%	0%	0%	0%	0%	80.0%
chi-square		19.792	31.510	17.976	17.778	0.816	31.510
*p*		**0.0001**	**0.0001**	**0.0001**	**0.0001**	0.846	**0.0001**

Bold indicates results with statistical significance.

**Table 4:** Approval of pregnancy terminations in case of lethal fetal defects vs. approval of other indications for terminations (N = 40).

Do You Approve of Pregnancy Termination in Case of Lethal Fetal Defects?		Severe Fetal Defects	Lethal Fetal Defects	Serious Maternal Disease	Pregnancy Being a Result of Rape	Unwanted Pregnancy	Never
yes, always and until the end of pregnancy	N	15	18	16	15	3	1
	%	48.4%	51.4%	50.0%	50.0%	60.0%	20.0%
yes, until the end of 12 gestational weeks	N	0	1	1	0	0	0
	%	0%	2.9%	3.1%	0%	0%	0%
yes, up to 22 gestational weeks	N	16	16	15	15	2	1
	%	51.6%	45.7%	46.9%	50.0%	40.0%	20.0%
no	N	0	0	0	0	0	3
	% z	0%	0%	0%	0%	0%	60.0%
chi-square		16.493	22.733	13.181	13.746	0.768	22.733
*p*		**0.001**	**0.0001**	0.004	0.003	0.857	**0.0001**

Bold indicates results with statistical significance.

**Table 5:** Readiness to perform a pregnancy termination personally depending on indications.

Indications for Abortion	Approval	No Approval
Abortion without medical indications	5 (12.5%)	30 (75.0%)
Severe fetal defects	30 (75.0%)	5 (12.5%)
Lethal fetal defects	34 (85%)	1 (2.5%)
Rape	30 (75.0%)	10 (12.5%)
Serious life-threatening maternal disease	31 (77.5%)	9 (22.5%)

**Table 6:** Approval of pregnancy termination in case of lethal defects vs. opinion on the possibility of termination in case of lethal defects in one’s own child.

			Would You Agree to Pregnancy Termination in Case of a Potential Lethal Defect Diagnosed in Your Child?				
			Strongly Agree	Agree	No Opinion	Disagree	Strongly Disagree
Do you approve of pregnancy termination in case of lethal fetal defects?	yes, always and until the end of pregnancy	N	13	4	1	0	0
		%	33.3%	10.3%	2.6%	0%	0%
	yes, up to 22 gestational weeks	N	7	5	1	3	1
		%	17.9%	12.8%	2.6%	7.7%	2.6%
	yes, up to 12 gestational weeks	N	0	0	1	1	0
		%	0%	0%	2.6%	2.6%	0%
	no	N	0	0	0	0	3
		%	0%	0%	0%	0%	7.7%
chi-square			42.119				
*p*			**0.0001**				

Bold indicates results with statistical significance.

**Table 7:** Approval of pregnancy termination in case of a threat to maternal life vs. opinion on the possibility of abortion of one’s own child if maternal life was threatened.

			Would You Agree to an Abortion of Your Child if Maternal Life Was Threatened?				
			Strongly Agree	Agree	No Opinion	Disagree	Strongly Disagree
Do you approve of pregnancy termination in case of a serious disease threatening maternal life?	yes, always and until the end of pregnancy	N	10	2	2	0	0
		%	26.3%	5.3%	5.3%	0%	0%
	yes, up to 22 gestational weeks	N	6	8	2	0	0
		%	15.80%	21.1%	5.3%	0%	0%
	yes, until the end of 12 gestational weeks	N	0	4	1	0	0
		%	0%	10.5%	2.6%	0%	0%
	no	N	0	0	0	1	2
		%	0%	0%	0%	2.6%	5.3%
chi-square			48.218				
*p*			**0.0001**				

Bold indicates results with statistical significance.

## References

[1] Higgins J.A., Schmuhl N.B., Wautlet C.K., Rice L.W. (2021). The Importance of Physician Concern and Expertise in Increasing Abortion Health Care Access in Local Contexts. Am. J. Public Health.

[2] Turk J., Landy U., Preskill F., Adler A., Steinauer J. (2021). The Integration of Abortion into Obstetrician-Gynecologists’ Practice after Comprehensive Family Planning Resident Training. Contraception.

[3] Zaręba K., Wójtowicz S., Banasiewicz J., Herman K., Jakiel G. (2021). The Influence of Abortion Law on the Frequency of Pregnancy Terminations-A Retrospective Comparative Study. Int. J. Environ. Res. Public Health.

[4] Bhakuni H., Miotto L. (2021). Conscientious Objection to Abortion in the Developing World: The Correspondence Argument. Dev. World Bioeth.

[5] Dickens B.M. (2021). Conscientious Objection and the Duty to Refer. Int. J. Gynaecol. Obs..

[6] Fink L.R., Stanhope K.K., Rochat R.W., Bernal O.A. (2016). “The Fetus Is My Patient, Too”: Attitudes Toward Abortion and Referral Among Physician Conscientious Objectors in Bogotá, Colombia. Int. Perspect. Sex. Reprod. Health.

[7] Harris L.F., Awoonor-Williams J.K., Gerdts C., Gil Urbano L., González Vélez A.C., Halpern J., Prata N., Baffoe P. (2016). Development of a Conceptual Model and Survey Instrument to Measure Conscientious Objection to Abortion Provision. PLoS ONE.

[8] Ramón Michel A., Kung S., López-Salm A., Ariza Navarrete S. (2020). Regulating Conscientious Objection to Legal Abortion in Argentina: Taking into Consideration Its Uses and Consequences. Health Hum. Rights.

[9] Wicclair M.R. (2000). Conscientious Objection in Medicine. Bioethics.

[10] Raus K., Mortier E., Eeckloo K. (2018). In Defence of Moral Pluralism and Compromise in Health Care Networks. Health Care Anal..

[11] Polish Government (1993). Act of January 7, 1993 on Family Planning, Protection of the Human Fetus and Conditions of Acceptability of Termination of Pregnancy. https://isap.sejm.gov.pl/isap.nsf/download.xsp/WDU19930170078/U/D19930078Lj.pdf.

[12] (2021). Trybunał Konstytucyjny Wyrok Trybunału Konstytucyjnego z 22 Października 2020 r. w Sprawie Art. 4a ust. 1 pkt 2 Ustawy z Dnia 7 Stycznia 1993 r. o Planowaniu Rodziny, Ochronie Płodu Ludzkiego i Warunkach Dopuszczalności Przerywania Ciąży (Dz. U. Nr 17, poz. 78, z 1995 r. Nr 66, poz. 334, z 1996 r. Nr 139, poz. 646, z 1997 r. Nr 141, poz. 943 i Nr 157, poz. 1040, z 1999 r. Nr 5, poz. 32 oraz z 2001 r. Nr 154, poz. 1792). https://trybunal.gov.pl/postepowanie-i-orzeczenia/wyroki/art/11300-planowanie-rodziny-ochrona-plodu-ludzkiego-i-warunki-dopuszczalnosci-przerywania-ciazy.

[13] Karolina Kropiwiec. Całkowity Zakaz Aborcji—Projekt Odrzucony w Pierwszym Czytaniu. Infor 2021. https://www.infor.pl/prawo/nowosci-prawne/5371711,Calkowity-zakaz-aborcji-projekt-odrzucony-w-pierwszym-czytaniu.html.

[14] Kasstan B. (2018). Irish Voters Repealed the Eighth: Now It’s Time to Ensure Access to Abortion Care in Law and in Practice. Reprod. Health Matters.

[15] Fetal Medicine Foundation (2021). FMF Accredited Practitioners NT Specialists. https://fetalmedicine.org/lists/map/certified/NT-specialist.

[16] Polish Gynaecological Society Polish Gynaecological Society Guideline on Prenatal Diagnosis 2009. https://www.google.com/url?sa=t&rct=j&q=&esrc=s&source=web&cd=&cad=rja&uact=8&ved=2ahUKEwiB6NeXo7n1AhWm-yoKHeMHAN0QFnoECAQQAQ&url=https%3A%2F%2Fjournals.viamedica.pl%2Fginekologia_polska%2Farticle%2Fdownload%2F46618%2F33405&usg=AovVaw0RWz1H5W6R5UwYB_T5W6_Y.

[17] Borowski D., Pietryga M., Basta P., Cnota W., Czuba B., Dubiel M., Fuchs T., Huras H., Iciek R., Jaczynska R. (2020). Practice Guidelines of the Polish Society of Gynecologists and Obstetricians—Ultrasound Section for Ultrasound Screening in Uncomplicated Pregnanc—2020. Ginekol. Pol..

[18] Hansen H. (1978). Brief Reports Decline of Down’s Syndrome after Abortion Reform in New York State. Am. J. Ment. Defic..

[19] Taylor M., Spillane A., Arulkumaran S.S. (2020). The Irish Journey: Removing the Shackles of Abortion Restrictions in Ireland. Best Pract. Res. Clin. Obstet. Gynaecol..

[20] Küng S.A., Wilkins J.D., de León F.D., Huaraz F., Pearson E. (2021). “We Don’t Want Problems”: Reasons for Denial of Legal Abortion Based on Conscientious Objection in Mexico and Bolivia. Reprod. Health.

[21] Schmuhl N.B., Rice L.W., Wautlet C.K., Higgins J.A. (2021). Physician Attitudes about Abortion and Their Willingness to Consult in Abortion Care at a Midwestern Academic Medical Center. Contraception.

[22] Saengruang N., Cetthakrikul N., Kulthanmanusorn A., Chotchoungchatchai S., Pudpong N., Suphanchaimat R. (2021). Self-Assessment of Attitudes towards Conditions to Provide Safe Abortion among New Medical Graduates in Thailand, 2018: An Application of Cross-Sectional Survey with Factor Analysis. BMC Womens Health.

[23] Roets E., Dierickx S., Deliens L., Chambaere K., Dombrecht L., Roelens K., Beernaert K. (2021). Healthcare Professionals’ Attitudes towards Termination of Pregnancy at Viable Stage. Acta Obstet. Gynecol. Scand..

[24] Lucchetti G., de Oliveira L.R., Leite J.R., Lucchetti A.L.G. (2014). SBRAME Collaborators Medical Students and Controversial Ethical Issues: Results from the Multicenter Study SBRAME. BMC Med. Ethics.

[25] Daniel S., Schulkin J., Grossman D. (2021). Obstetrician-Gynecologist Willingness to Provide Medication Abortion with Removal of the in-Person Dispensing Requirement for Mifepristone. Contraception.

[26] McLeod C., Javlekar A., Flink-Bochacki R. (2021). Exploring the Relationship between Abortion Provision and Providers’ Personal Pregnancy and Parenting Experiences. Womens Health Issues.

[27] Bento S.F., de Pádua K.S., de Pacagnella R.C., Fernandes K.G., Osis M.J.D., Duarte G.A., Faúndes A. (2020). Advantages and Disadvantages of Medical Abortion, According to Brazilian Residents in Obstetrics and Gynaecology. Rev. Bras. Ginecol. Obstet..

[28] Biggs M.A., Casas L., Ramm A., Baba C.F., Correa S.P. (2020). Medical and Midwifery Students’ Views on the Use of Conscientious Objection in Abortion Care, Following Legal Reform in Chile: A Cross-Sectional Study. BMC Med. Ethics.

[29] Casas L., Freedman L., Ramm A., Correa S., Baba C.F., Biggs M.A. (2020). Chilean Medical and Midwifery Faculty’s Views on Conscientious Objection for Abortion Services. Int. Perspect. Sex. Reprod. Health.

[30] Magelssen M., Le N.Q., Supphellen M. (2019). Secularity, Abortion, Assisted Dying and the Future of Conscientious Objection: Modelling the Relationship between Attitudes. BMC Med. Ethics.

[31] Lappen J.R., Vricella L.K., Andrews V., Christensen E., Heuser C.C., Horvath S., Johnson C.T., Louis J.M., Luchowski A.T., Society for Maternal-Fetal Medicine (2021). Society for Maternal-Fetal Medicine Special Statement: Maternal-Fetal Medicine Subspecialist Survey on Abortion Training and Service Provision. Am. J. Obstet. Gynecol..

[32] Zaręba K., La Rosa V.L., Kołb-Sielecka E., Ciebiera M., Ragusa R., Gierus J., Commodari E., Jakiel G. (2020). Attitudes and Opinions of Young Gynecologists on Pregnancy Termination: Results of a Cross-Sectional Survey in Poland. Int. J. Environ. Res. Public Health.

[33] Meyers C., Woods R.D. (1996). An Obligation to Provide Abortion Services: What Happens When Physicians Refuse?. J. Med. Ethics.

[34] O’Connor R., O’Doherty J., O’Mahony M., Spain E. (2019). Knowledge and Attitudes of Irish GPs towards Abortion Following Its Legalisation: A Cross-Sectional Study. BJGP Open.

[35] Power S., Meaney S., O’Donoghue K. (2021). Fetal Medicine Specialist Experiences of Providing a New Service of Termination of Pregnancy for Fatal Fetal Anomaly: A Qualitative Study. BJOG.

[36] Bennett A.H., Freedman L., Landy U., Langton C., Ly E., Rocca C.H. (2020). Interprofessional Abortion Opposition: A National Survey and Qualitative Interviews with Abortion Training Program Directors at U.S. Teaching Hospitals. Perspect. Sex. Reprod. Health.

[37] Qian J.-L., Pan P.-E., Wu M.-W., Zheng Q., Sun S.-W., Liu L., Sun Y.-P., Yu X.-Y. (2021). The Experiences of Nurses and Midwives Who Provide Surgical Abortion Care: A Qualitative Systematic Review. J. Adv. Nurs..

[38] Głusiec W. (2022). Hospital Chaplains as Ethical Consultants in Making Difficult Medical Decisions. J. Med. Ethics.

